# Housing and husbandry factors affecting zebrafish novel tank test responses: a global multi-laboratory study

**DOI:** 10.1038/s41684-025-01548-x

**Published:** 2025-05-26

**Authors:** Courtney Hillman, Barbara D. Fontana, Tamara G. Amstislavskaya, Maria A. Gorbunova, Stefani Altenhofen, Karissa Barthelson, Leonardo M. Bastos, João V. Borba, Carla D. Bonan, Caroline H. Brennan, Amaury Farías-Cea, Austin Cooper, Jamie Corcoran, Eduardo R. Dondossola, Luis M. Martinez-Duran, Matheus Gallas-Lopes, David S. Galstyan, Ella O. Garcia, Ewan Gerken, Robert Hindges, Justin W. Kenney, Maxim A. Kleshchev, Tatiana O. Kolesnikova, Adele Leggieri, Sergey L. Khatsko, Michael Lardelli, Guilherme Lodetti, Giulia Lombardelli, Ana C. Luchiari, Stefani M. Portela, Violeta Medan, Lirane M. Moutinho, Evgeny V. Nekhoroshev, Barbara D. Petersen, Maureen L. Petrunich-Rutherford, Angelo Piato, Maurizio Porfiri, Emily Read, Cássio M. Resmim, Eduardo P. Rico, Denis B. Rosemberg, Murilo S. de Abreu, Catia A. Salazar, Thailana Stahlhofer-Buss, Júlia R. Teixeira, Ana M. Valentim, Alexander V. Zhdanov, Patricio Iturriaga-Vásquez, Xian Wang, Ryan Y. Wong, Allan V. Kalueff, Matthew O. Parker

**Affiliations:** 1 Surrey Sleep Research Centre, Department of Clinical and Experimental Medicine, School of Biosciences, University of Surrey, Surrey, UK; 2 Laboratory of Experimental Neuropsychobiology, Department of Biochemistry and Molecular Biology, Natural and Exact Sciences Center, Federal University of Santa Maria, Santa Maria, Brazil; 3 Department of Biological Sciences, Wayne State University, Detroit, MI USA; 4 Laboratory of Experimental Models of Neuropsychiatric Disorders, Scientific Research Institute of Neurosciences and Medicine, Novosibirsk State University, Novosibirsk, Russia; 5 Ural Federal University, Yekaterinburg, Russia; 6 Laboratório de Neuroquímica e Psicofarmacologia, Escola de Ciências da Saúde e da Vida, Pontifícia Universidade Católica do Rio Grande do Sul, Porto Alegre, Brazil; 7 Alzheimer’s Disease Genetics Laboratory, School of Biological Sciences, Faculty of Sciences, Engineering and Technology, The University of Adelaide, Adelaide, South Australia Australia; 8 Childhood Dementia Research Group, College of Medicine and Public Health, Flinders Health and Medical Research Institute, Flinders University, Adelaide, South Australia Australia; 9 Laboratório de Psicofarmacologia e Comportamento, Departamento de Farmacologia, Instituto de Ciências Básicas da Saúde, Universidade Federal do Rio Grande do Sul, Porto Alegre, Brazil; 10 School of Biological and Behavioural Sciences, Queen Mary University of London, London, UK; 11 Molecular Pharmacology and Medicinal Chemistry Lab, Facultad de Ingeniería y Ciencias, Universidad de la Frontera, Temuco, Chile; 12 Department of Psychology, University of Nebraska at Omaha, Omaha, NE USA; 13 Translational Psychiatry Laboratory, Graduate Program in Health Sciences, University of Southern Santa Catarina, Criciúma, Brazil; 14 St. Petersburg State University, St. Petersburg, Russia; 15 Department of Psychology, Indiana University Northwest, Gary, IN USA; 16 Centre for Developmental Neurobiology and MRC Centre for Neurodevelopmental Disorders, King’s College London, London, UK; 17 Neurobiology Program, Sirius University of Science and Technology, Sochi, Russia; 18 Department of Mechanical and Aerospace Engineering, Department of Biomedical Engineering, Center for Urban Science and Progress, New York University, Tandon School of Engineering, New York, NY USA; 19 FishLab, Department of Physiology and Behavior, Federal University of Rio Grande do Norte, Natal, Brazil; 20 Instituto de Fisiología, Biología Molecular y Neurociencias, Consejo Nacional de Investigaciones Científicas y Tecnológicas, Facultad de Ciencias Exactas y Naturales, Universidad de Buenos Aires, Buenos Aires, Argentina; 21 Graduate Program in Health Sciences, Federal University of Health Sciences of Porto Alegre, Porto Alegre, Brazil; 22 Western Caspian University, Baku, Azerbaijan; 23 The International Zebrafish Neuroscience Research Consortium, Slidell, LA USA; 24 Laboratory Animal Science, Instituto de Investigação e Inovação em Saúde, Universidade do Porto, i3S, Porto, Portugal; 25 Department of Biology, University of Nebraska at Omaha, Omaha, NE USA; 26 Department of Biosciences and Bioinformatics, School of Science, Xi’an Jiaotong-Liverpool University, Suzhou, China; 27 Suzhou Municipal Key Laboratory of Neurobiology and Cell Signaling, School of Science, Xi’an Jiaotong-Liverpool University, Suzhou, China

**Keywords:** Scientific community, Research data, Neuroscience, Psychology

## Abstract

The reproducibility crisis in bioscience, characterized by inconsistent study results, impedes our understanding of biological processes. Global collaborative studies offer a unique solution to this problem. Here, we present a global collaboration using the zebrafish (*Danio rerio*) novel tank test, a popular behavioral assay for anxiety-like responses. We analyzed data from 20 laboratories worldwide, focusing on housing conditions and experimental setups. Our study included 488 adult zebrafish, tested for 5 min, focusing on a variety of variables. Key findings show that female zebrafish exhibit more anxiety-like behavior than males, highlighting sex as a critical variable. Housing conditions, including higher stocking densities and specific feed types, also influenced anxiety levels. Optimal conditions (5 fish/L) and nutritionally rich feeds (for example, rotifers) mitigated anxiety-like behaviors. Environmental stressors, such as noise and transportation, significantly impacted behavior. We recommend standardizing testing protocols to account for sex differences, optimal stocking densities, nutritionally rich feeds and minimizing stressors to improve the reliability of zebrafish behavioral studies.

## Abstract (web summary)

This Article presents the results of a global multi-laboratory study assessing the effects of housing conditions and experimental settings on novel tank test responses in adult zebrafish.

## Main

The growing data repeatability crisis in bioscience, marked by the frequent failure of studies to produce consistent results upon replication^1–7^, not only represents a substantial reputational challenge but also limits progress in understanding basic biological processes^5,8–10^. The causes of this crisis are broad and multifaceted, including selective reporting, publication bias, incomplete data reporting and, critically, variability in experimental methods and laboratory conditions^10–14^. One potential solution to this problem is global collaborative studies, in which multiple laboratories worldwide examine the same research question using diverse experimental setups and testing protocols^5^. By systematically assessing the influence of different between-laboratory parameters on a single outcome measure, we can enhance the robustness and generalizability of findings, helping clarify factors that impact study outcomes.

The zebrafish (*Danio rerio*) is a powerful vertebrate model widely used to understand and characterize the biology of a range of neuropsychiatric disorders, including anxiety-related disorders^15–19^. Anxiety disorders rank as the most prevalent mental health conditions globally, affecting over 300 million individuals^20^. Exacerbated anxiety is common as a comorbid condition in individuals diagnosed with psychiatric disorders such as major depression^21^, bipolar disorder^22^ and schizophrenia^23^.

One of the most popular behavioral tests to study anxiety-like responses in adult zebrafish is the novel tank (or novel tank diving) test based on the innate defensive geotaxis of zebrafish. Its popularity stems from its increased sensitivity compared with other common behavioral assays, such as the light/dark response^24^. Recent research has attempted to determine the neuronal basis of the zebrafish novel tank response, with imaging analyses suggesting an integral role of the ventral telencephalon and the anterior parvocellular preoptic nucleus^25^. Screening the literature using the search terms (‘novel tank’ OR ‘tank diving’ OR ‘geotaxis’) AND ‘zebrafish’ in the PubMed database (https://pubmed.ncbi.nlm.nih.gov/, accessed 24 May 2024), we identified 354 records published between 2007 (when the first publication using this protocol appeared (ref. ^26^)) and May 2024, which is an article on average every 2–3 weeks. These studies range from toxicity assessment^26–31^ to the characterization and understanding of psychiatric disorders^32–38^(Supplementary Fig. 1). Any reviews, preprints and original research not using zebrafish or the novel tank test were excluded from analyses (*n* = 17), leaving 337 original relevant research articles. The results of this search confirmed the wide usage of the novel tank tests but revealed substantial variability in experimental setup and conditions.

The novel tank test exploits the natural tendency of the fish to swim to the bottom of a novel environment, followed by a gradual habituation and increased exploration over time, usually 5–6 min (refs. ^26,33,39^). The standard response of zebrafish typically involves an initial phase where animals spend most of the time in the bottom and can show increased anxiety responses, such as freezing (immobility). This behavior is then often followed by a habituation phase where fish gradually increase their activity levels and start to explore the more ‘dangerous’ areas of the tank (that is, the top half or third, which in nature is a more susceptible predation zone)^26,33,40–42^. Typically, one of the two endpoints used is either the time spent in the top portion of the tank divided in three^26,43–45^ or two zones^33,39,46–48^, or the number of entries into the top zone^33,39,47^. Often both metrics—the time spent in top as a measure of overall anxiety-like behavior and the number of top entries—can be used as a measure of exploratory tendency^33,41,49^. Although zebrafish swim in a three-dimensional space, both two- and three-dimensional approaches are utilized for behavioral scoring of the animals. This may lead to inaccurate or unreproducible reporting of individual and social behavior, undermining data integrity (especially related to zebrafish general locomotion), and a possible overestimate of the number of animals required for the studies^50^.

Despite its wide usage in zebrafish neurobehavioral research, there is little consensus concerning the optimal conditions for this task, such as the test tank size, lighting level and tank color, among several other factors. In addition, there is no information about how housing and husbandry conditions might affect performance and endpoints. Indeed, pretest housing can strongly affect behavioral performance. For example, housing fish in a tank of the same size as the tank in which they are tested reduces the novel tank diving effects^28^. In addition, tank diving effects are mediated by pretesting group size, with fish housed individually or two in a tank before testing showing a less extreme response to novelty compared with group-housed fish; sex ratios in housing tanks also substantially impact the novel tank behavior^51,52^.

However, the lack of reporting and consistency in the testing conditions of the novel tank test raises questions about how much the tank setup, as well as other environmental factors related to housing conditions, can affect the responses of fish in this test. Therefore, in the present study, 20 laboratories from across the world performed the same experiment to assess the effects of housing conditions and experimental settings on adult zebrafish (*n* = 24 (12 male and 12 females)) responses to the standard 5-min novel tank test.

## Results

### Data distribution and differences across laboratories

**Fig. 1:**
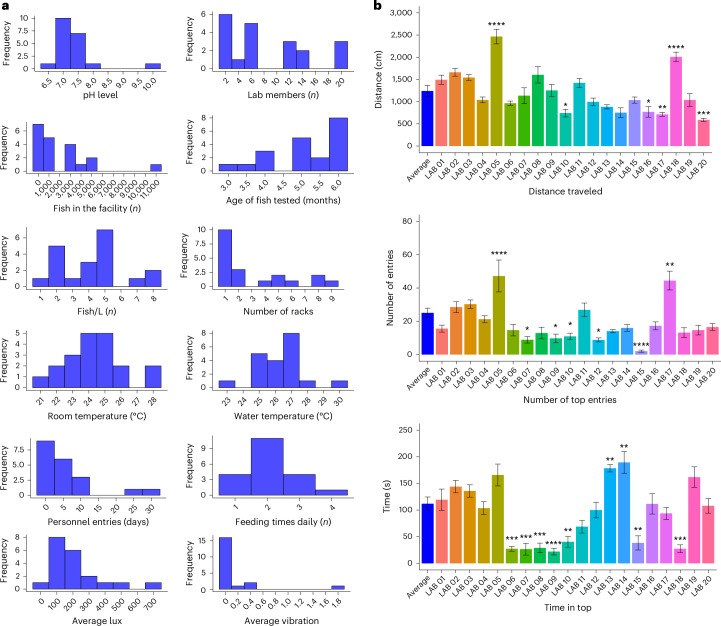
Comparative analysis of quantitative parameters across laboratories. **a**, The distribution of quantitative parameters across laboratories. **b**, Differences in main variables comparing each laboratory with the average of the data. Data are represented as mean ± s.e.m. and analyzed by ANOVA followed by Dunnett’s post-hoc test. Asterisks indicate statistical differences compared with the average (*P *<* 0.05, **P *<* 0.01, ***P *<* 0.001 and ****P *<* 0.0001).

The dataset used in the present study consisted of a total of 2,435 observations and 47 variables, including both categorical and continuous predictors (https://osf.io/4chwt/?view_only=024aa4208a83420c8fa38e2e0c64943a). Initial exploratory data analysis was performed to examine distributions of continuous predictors (Fig. 1a), followed by an analysis of variance (ANOVA) and Dunnett’s post-hoc comparison test to evaluate significant differences between the laboratories (Fig. 1b). We found that all behavioral parameters (mean distance traveled, time in top and number of entries to the top) varied between the laboratories, with mean distance traveled and number of entries significantly increased for two of the collaborators (****P < 0.0001; the same collaborators) and decreased for others (*P < 0.05) compared with the average. Notably, the number of entries was found to be higher in two laboratories and lower in another five (*P < 0.05). Similarly, two laboratories showed an increase in time spent on top (*P < 0.05), a parameter usually associated with reduced anxiety-like behavior, while another seven laboratories showed the opposite (*P < 0.05). This result demonstrates the variability of behavioral parameters across the fish tested in these 20 groups. The changes in time spent in top and number of entries across time bins are shown in Supplementary Fig. 2. We also ran Spearman’s correlations on the average scores per fish (that is, over the full 5-min exposure) to examine the intercorrelation of outcome variables and distance traveled and to identify initial patterns across the sample. There was a weak positive correlation between distance traveled during the test and time spent in the top of the tank (s) (*ρ* = 0.14, **P = 0.002), and a moderate positive correlation between distance traveled during the test and number of top entries (*ρ* = 0.45, ****P < 0.001). There was also a moderate positive correlation between time in the top (s) and number of top entries (*ρ* = 0.52, ****P < 0.001). Scatter plots are shown in Supplementary Fig. 3.

**Fig. 2:**
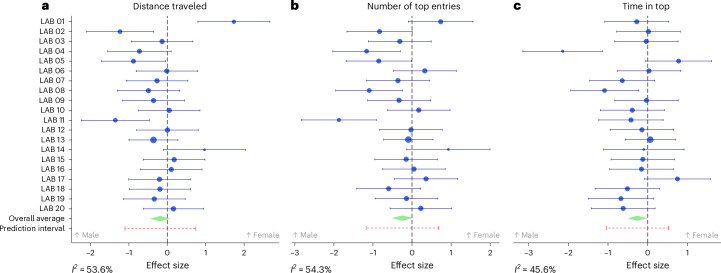
Forest plots of sex differences in behavioral metrics across laboratories. **a**–**c**, Forest plots illustrating the effect size of distance traveled (**a**), entries to the top zone (**b**) and time in the top zone (**c**) for various laboratories when comparing females and males. A positive effect size indicates higher values for females, while a negative effect size indicates higher values for males. Each row represents a different laboratory, with the overall estimate shown at the bottom (green diamonds), calculated using a random-effects method. The size of each circle is proportional to the weights of each effect size of each laboratory, and the arrows indicate the CIs of each laboratory effect size.

We next considered overall sex differences, as this variable has already been reported to affect zebrafish anxiety- and activity-related behavioral endpoints (see above). Figure 2 shows forest plots representing the effect sizes across laboratories, when comparing females and males. We found no significant effect of sex on distance traveled (−0.19, 95% confidence interval (CI) −0.46 to 0.08; Fig. 2a), entries to the top (−0.25, 95% CI −0.52 to 0.03; Fig. 2b) or time spent in the top zone (−0.26, 95% CI −0.51 to −0.01; Fig. 2c).

Based on the initial descriptive analysis, the dataset showed moderate heterogeneity (*I*^2^) values of 53.62, 54.28 and 45.61 for the effect size of distance traveled, entries to the top zone and time spent in the top zone, respectively. To better understand these discrepancies and identify the most influential predictors, we next used Lasso regression, which helps in selecting and regularizing variables to improve model accuracy and interpretability.

### Analysis of variability in laboratory conditions and experimental settings

To understand how laboratory conditions and experimental settings influence data variability, we performed Lasso regression, chosen here as it effectively handles high-dimensional data (that is, datasets with many predictors) and selects relevant predictors by shrinking less important coefficients to zero, thereby enhancing the model’s interpretability and predictive accuracy despite the diverse (and potentially collinear) variables present in the dataset. Mixed-effects models were first fit to account for the hierarchical structure of the data, with random effects for ‘Lab ID’ nested within ‘Fish ID’. Residuals from these models were extracted for time in top (s) and the number of top entries to be used as response variables, and distance traveled was used as a covariate in subsequent analyses. Design matrices were created for different sets of fixed effects, including main effects, sex, time and sex × time interactions. Lasso regression models were fit, and cross-validation was used to determine the optimal lambda values to prevent overfitting (Supplementary Fig. 4).

To examine model fit, we calculated the deviance explained by each model, and to examine model parsimony, we calculated the Akaike information criterion (AIC; Table 1).

**Table 1:** Deviance explained and comparison of AIC for different models

Model	Deviance explained (time top)	Deviance explained (number top entries)	AIC (time top)	AIC (number top entries)
Null	NA	NA	18,466.26	12,885.29
Main	2.83	12.19	18,398.98	12,607.48
Sex interaction	3.13	12.66	18,414.79	12,618.59
Time interaction	6.90	18.33*	18,371.70*	12,444.37*
Sex × time interaction	7.33*	18.04	18,441.16	12,506.38

NA, not applicable. *P *<* 0.05.

Based on the values, the time interaction model was selected for both time in top (s) and number of top entries, as it had the lowest AIC and the highest deviance explained for number of top entries, as well as the lowest AIC and a high deviance explained for the time in top, indicating the best model for both fit and parsimony. Bootstrapping with 1,000 iterations was then performed for each model to estimate the variability of the coefficients and generate 95% CIs (Supplementary Fig. 5).

For time spent in the top (s), the time interaction model (deviance explained 6.9%, optimal lambda 0.01) revealed several nonzero predictors including sex, density (number of fish/L in the housing room), total number of racks and whether the fish were fed rotifer diets. There were also several nonzero interactions with time, including the pH, number of fish/L, number of racks in the housing room, whether the racks were static or circulating, total number of fish, water temperature, lux levels, vibration, noise levels, wall color and tank color, number of laboratory members, personnel entries, behavior testing in a separate room, feed types and various sex × time interactions as significant predictors. Similarly, for the number of top entries model (deviance explained 18.33%, optimal lambda 0.01), nonzero predictors in the time interaction model included sex, number of fish/L, whether the racks were circulating, total number of fish, vibration, noise levels, tank and floor color, gender of the personnel, feed types and times, and various time interactions. Finally, significant predictors for both time spent in the top and number of top entries were calculated on the basis of 95% CI not including 0, and these are shown in Supplementary Fig 6.

For the time spent in the top of the tank (s), the models revealed several main effects and interactions (Supplementary Fig 6). In terms of main effects, once we controlled for random effects (and as we saw suggested in the overall models), female fish spent less time in the top section compared with males (Supplementary Fig. 5a). In addition, the use of rotifer feed was associated with more time spent at the top. A greater number of racks in the facility was associated with less time spent in the top section, whereas a higher density of fish/L was associated with more time spent at the top. Several interaction effects were observed, which identified factors that changed their influence over the 5-min exposure time (Supplementary Fig. 5b). First, there were two factors that were associated with a negative effect over time (that is, the variable influence gradually decreased for time spent in top of the tank). For example, while the presence of rotifer feed initially increased the time spent at the top, this effect diminished over time, as indicated by a negative interaction between rotifer feed and time. Similarly, the number of fish in the facility was initially predictive of more time spent in the top, but this effect weakened over time. Other interactions showed a positive effect over time (that is, had an increasing effect the more time the fish spent in the top of the tank), including pH and whether they were transported to another room for testing (Supplementary Fig. 5b).

For the number of top entries, the models identified that a higher density of fish/L yielded more top entries, suggesting more exploration. Higher overall noise in the aquarium was associated with fewer top entries (Supplementary Fig 5c). However, this effect of noise was time dependent, with the interaction of noise and time in the test showing a positive effect over time (that is, noise had a stronger effect on top entries as time went on; Supplementary Fig 5d).

Finally, the significant predictors identified by the Lasso regression were then used to construct new models to validate their influence on the original linear mixed models (LMMs). For the time in top parameter, these models included sex, number of racks in the facility, number of fish/L, whether the fish were fed rotifers, the number of personnel entries, the pH of the housing room and, finally, whether behavior testing was carried out in a separate room. All were run as interactions with time as an interaction factor (Table 2). Next, for the number of top entries model, variables chosen were number of fish/L and noise (dB) in the housing room, both interacting with time (Table 3).

**Table 2:** LMMs for time spent in top with predictors from Lasso regression

Predictor	Estimate	s.e.m.	d.f.	*t* value	Pr(>|t|)
(Intercept)	21.72	6.52	14.71	3.33	0.005**
Sex	1.63	0.88	1,526.02	1.87	0.062
Number of racks	−2.49	1.15	14.77	−2.16	0.047*
Fish/L	1.79	1.58	14.84	1.13	0.276
Rotifer feed	10.22	8.52	14.67	1.2	0.249
Time	4.46	2.77	1,971.6	1.61	0.107
Separate room for performing behavior	−5.83	6.58	14.65	−0.89	0.390
Time × rotifer feed	3.98	1.83	1,971.66	2.17	0.030*
Time × number of fish	−0.06	0.25	1,971.76	−0.24	0.81022
Time × personnel entries	0.02	0.55	1,971.73	0.05	0.96415
Time × pH	−0.66	0.38	1,971.6	−1.76	0.07911
Time × separate room	2.03	0.62	1,971.84	3.28	0.00105**

Time spent in top measured in seconds. *P < 0.05, **P < 0.01.

**Table 3:** LMMs for number of top entries with predictors from Lasso regression

Predictor	Estimate	s.e.m.	d.f.	*t* value	Pr(>|t|)
(Intercept)	2.47	1.25	16.72	1.98	0.064
Fish/L	0.30	0.27	16.83	1.12	0.279
Noise (dB)	0.31	0.52	16.73	0.60	0.556
Time	0.97	0.07	1,983	13.64	<2 × 10^−16^***
Time × noise (dB)	0.19	0.07	1,983	2.67	0.007**

**P *<* 0.01, ****P *<* 0.0001.

**Fig. 3:**
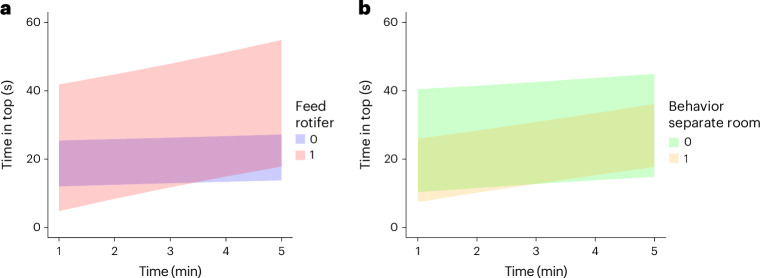
Effects of rotifer feeding and transport on time spent in the top of the tank. **a**, The interaction between rotifer feed (1 = yes, 0 = no) and time (minutes 1–5 in the test) for time spent in the top of the tank (s). Fish that were fed rotifers (1) showed a more rapid increase in time spent in the top compared with those that were not (0) throughout the test period. **b**, The interaction between transport before testing (1 = yes, 0 = no) and time (minutes 1–5 in the test) for time spent in the top of the tank (s). Fish tested in the same room where they were housed (0) spent more time in the top overall. However, those transported to a separate testing room (1) showed a gradual increase in time spent in the top, finishing the testing period with a similar amount of time in the top as those tested in their home tank room (0). Sixteen laboratories performed the test in a separate room, and two laboratories fed rotifers.

Validation of the Lasso regression predictors with the original LMMs revealed several variables that significantly affected both aspects of tank diving performance (time in top and number of entries; Fig. 3). These included the overall size of the facility, which was associated with less time spent in the top overall, the presence of rotifers in the diet (which was associated with more time in the top, indicative of lower levels of anxiety-like behavior) and whether the fish were transported to the behavior testing room, which was also associated with less time in the top (that is, increased anxiety-like behavior). In addition, the impact of noise on behavioral outcomes in the novel tank test was confirmed (Table 3). However, both latter effects (the rotifers and transport) were significant only in the interaction with time. Figure 3 characterizes the interaction effects.

## Discussion

The present study sought to examine the factors that underlie variability in adult zebrafish behavior across different laboratories, concentrating on one of the most widely used protocols for measuring anxiety-like behavior, the novel tank test. Predominantly a zebrafish-specific anxiety test, it has been used successfully with other fish species and holds excellent face validity likened to the rodent open-field test^41,53,54^. Our results highlight that the impact of laboratory-specific conditions and experimental settings on experimental outcomes in the novel tank test are relatively minor in nature. These factors generally do not strongly affect the outcome of the study, indicating that fish will tend to dive to the bottom of the novel environment and gradually rise to the top to explore over a 5-min period, regardless of experimental factors and differing environments. Thus, time in the test was critical across all laboratories. There was some variability in distance traveled, which varied between laboratories independently and did not correlate with other response variables (Fig. 1). However, we did find that the correlation between distance traveled and number of top entries was considerably stronger than between distance traveled and time spent in the top, suggesting that these measures should not be used indiscriminately interchangeably and perhaps that number of top entries is less indicative of anxiety-like behavior than time spent in the top (that is, it may be more indicative of general movement; Supplementary Fig. 3).

We used a Lasso regression to identify specific laboratory conditions and experimental settings that significantly influence responses in the novel tank test. We then validated these predictors with LMMs. Several predictors remained significant in the validation models, including type of feed, overall size of the facility, and environmental factors such as noise. Several interaction effects over time were observed, indicating that the factors affected habituation to the test^51^. In summary, some housing and husbandry conditions, such as specific feed types and not transporting the animals to another room before testing, reduced levels of anxiety-like behavior. Background noise was found to be the most significant variable affecting top entries, with higher noise levels leading to more top entries and this effect increasing over time. Noise is usually overlooked in fish studies but was recently shown to increase anxiety-like behavior in zebrafish^55^. Collectively, this intricate and complex interaction of factors with time confirms the critical importance of including time as a covariate in analyses, even if it is not treated as a main effect^51^.

Although the Lasso models identified sex differences—previously reported and observed in some laboratories (Fig. 2)—this was not confirmed in the validation models, suggesting inconsistency. Previous research indicates that female zebrafish tend to show higher levels of anxiety-like behavior than males due to a combination of behavioral, neurobiological, social and pharmacological factors^56–59^. However, the sex differences are shown to be parameter-dependent, with not all reported findings showing sex differences^60^. In addition, previous data suggest that female zebrafish have increased sensitivity to environmental stressors, in which social and predator stimuli elicit higher anxiety-like responses, also reinforcing the role of environmental cues in influencing anxiety-related phenotypes^61^. Reasons for these differential anxiety responses may rest in neurobiological differences^62^. For example, female zebrafish have higher glucose mobilization to hypothalamic brain areas and a distinct pattern of adrenoceptor expression compared with males, which might contribute to their heightened anxiety-like responses^63^. In general, there is an extensive literature on the importance of including sex differences in analyses of animal models of neuropsychiatric disorders^64^. Despite the variability between laboratories in sex effects, the findings further demonstrate the necessity of accounting for sex as a biological variable in behavioral research to ensure the robustness and reproducibility of experimental results.

Several other husbandry factors were associated with behavioral differences in novel tank test responses. Although these factors did not have a strong effect on the overall tank diving, they did explain some variance in these responses. For example, fish held at higher stocking densities spent more time in the top, suggesting lower anxiety-like behavior. Here, the data from the 20 participating laboratories show stock densities ranging from 1 to 8 fish/L, with an average of 3 and 4.75 fish/L for static and recirculating tanks, respectively. Although high stocking densities can lead to increased stress among zebrafish, this is only likely to occur at the extremes, which were not represented in our sample^51,65,66^. However, lower densities, while reducing physical crowding, can also lead to stress due to increased visibility of conspecifics, potentially increasing aggression and anxiety-like behavior^67^. Zebrafish at lower densities may exhibit higher cortisol levels and more aggressive behavior, which can be associated with pronounced anxiety-like responses, such as tank diving^65,66^. It is worth noting that, although there is a lack of reporting on the interaction between rack type (static or recirculating) and stocking density, it is likely that this has a significant effect on behavioral outcomes. Therefore, it is important to emphasize that very high or very low stocking density can lead to higher anxiety-like behaviors, but as has been previously reported, stocking densities of around 5 fish/L seem to be optimal in terms of the anxiety-like responses in zebrafish. We also report a significant effect of the size of facility on the time in the top, with an increased number of fish in the facility resulting in time-dependent heightened anxiety-like responses (reduced time in top; Supplementary Fig. 5b). Thus, it is apparent that the size of the facility and stocking densities have a critical role in zebrafish novel tank test behavior.

Feed type was an additional predictor, but the data were not clear. We observed that fish that were fed rotifers showed reduced anxiety-like responses. However, it is important to note that (although this was weighted in the statistical models) only two laboratories reported using this feed type, with one reporting feed during developmental stages and the other throughout fish life. Therefore, the extent to which rotifers impact larval development and subsequently novel tank test behavior remains a preliminary finding due to many fish being obtained commercially. However, rotifers, as well as other food sources, are a rich source of essential nutrients, including omega-3 and omega-6 fatty acids, proteins and vitamins, which could support overall health and reduce stress-related behaviors^68^. In addition, the provision of live feed ensures that zebrafish have access to a continuous and readily available food source, which stimulates the natural environment of fish^69^. This would probably reduce the stress associated with hunger and food competition, leading to lower levels of anxiety-like behavior^70,71^. Notably, introducing live food into an animal’s diet could serve as both nutritional and sensory enrichment^72^. However, interestingly, there was a time × rotifer interaction here, with the positive effects reducing as a function of time in the test, suggesting that fish were initially less stressed, but this state normalized over time. Thus, the constant presence of food in the maintenance environment could highlight the novelty of the test apparatus by leading to food seeking and consequently increasing anxiety over time. Collectively, these findings suggest that developmental feed type—specifically rotifers as no other commonly used feed type had an impact—may be important in predicting tank diving performance and should be accounted for in studies. This is a hypothesis at this stage, however, because we do not have sufficient evidence to suggest that any feeding regimen is preferable. Optimal zebrafish feed is very poorly understood, and the findings here further underscore the importance of working as a community to understand the best feeds for our animals^73^. This will be a critical area for future work aiming to elucidate how distinct feeding protocols influence anxiety in zebrafish research.

Transporting fish into a different room for behavioral testing was a significant predictor of time spent in the top of the tank. Evidence is clear that zebrafish respond strongly to novel environments. For example, zebrafish housed in a familiar environment exhibit lower baseline anxiety and cortisol levels, suggesting that familiar settings reduce stress and anxiety^74^. In addition, the presence of familiar cues, such as the same water from their home tank, can mitigate the anxiogenic effects of novel settings. For instance, zebrafish tested in home tank water show less anxiety-like behavior compared to those tested in novel water^75^. Although there is no direct evidence that this is the case in new rooms (that is, with novel extra-tank cues), it is possible that, similarly to tank water, novel testing environment also generalizes anxiety responses. In addition, this can be a potential limitation of the present study, as we did not ask participating laboratories to control for habituation time in the novel testing room. Thus, future research can examine this aspect directly. In addition to the novel environment, transport stress elevates cortisol levels in zebrafish, indicating an acute stress response, accompanied by increased glucose levels, which are secondary stress markers^76^. Again, this can represent a limitation here, as we did not ask laboratories for the timing of the transportation to the testing room. Given the observed effects of testing in a novel room, this could be a promising area for future research.

## Limitations

Although we identified numerous factors impacting behavioral outcomes in the novel tank test, several parameters were not included in the evaluation. While our study focused on husbandry parameters, we acknowledge the importance of other test-related parameters, and therefore we suggest a similar investigation to this one be performed related to test parameters. We have also provided in Supplementary Table 1 a comprehensive list of the parameters that must be reported on for the novel tank test. There may be other parameters of importance in addition to those included, and we reflect on some below.

While we asked laboratories whether behavioral testing was conducted in a separate room from housing facilities, we did not account for habituation time in the new environment, despite previous research illustrating its importance for behavioral evaluation^39,51^. In addition, Fontana et al.^77^ identified significant effects on novel tank test outcomes related to the number of water changes between different recordings.

Most of the data we collected were linked to the environmental conditions of the laboratory. However, some factors potentially affecting behavior during the behavioral recording of the novel tank test were not considered. For example, we did not account for water column depth. It is known that different background colors and light intensities can elicit varied responses in zebrafish anxiety assays^78^. Deeper tanks have also shown greater reliability, with less data variability and reduced anxiety-related changes in animals compared with shallower tanks^79^. The differences in water depth could influence data analysis by affecting the size of the top area of the tank. In addition, continuous measurement of the behavior (for example, distance from bottom) throughout the tank rather than analysis of time spent in the upper versus lower tank would increase test replicability and comparison of data across different conditions, therefore being a useful variable to include in future analyses.

We did not consider the breeding status of the fish, which can significantly impact behavior. Breeding, especially pair breeding, is a stressful event^47,51^, and if the novel tank test was conducted on recently bred fish, their behavior might differ from those never bred. The impact of feeding status on the day of testing, especially for those using flake foods, was also not accounted for^80^.

Water chemistry parameters were also not considered. Changes in water salinity and the presence of chemosensory cues from conspecifics substantially impact zebrafish behavior^81^. Additional limitations include the time of day of behavioral experimentation not being standardized, which can affect behavior. The dimensions of the novel tank used for behavioral assays were not standardized, which can influence the stress response. Some of the participating laboratories in this study measured swimming behavior in three dimensions and others in two dimensions, impacting metrics such as exploratory behavior and distance traveled.

The experimenter’s gender, age and frequency of working with fish were not controlled for. For example, in rodents, stress-like behaviors have been identified depending on experimenter identity^82^; however, zebrafish models may be more resilient to variation in experimenter identity^83^. The level of zebrafish behavioral experience in the laboratories, based on the principal investigator’s total years of experience, was also not considered.

We had initially intended to include the strain of fish used as a factor. However, several laboratories utilize commercially bought zebrafish, and use ‘outcrossed’ animals (in which the strain cannot accurately be determined), so the strain of zebrafish was not considered as a factor here. The use of both outbred populations and well-defined strains increases genetic diversity and may align more closely with natural populations and different settings used at laboratories across the world. Importantly, frequent inbreeding has previously been shown to significantly impact novel tank test behavior^33^, and as such, strain should at the very least be reported in studies (as is typical). Sex ratios during housing were not reported, and therefore the effect of an uneven sex split during housing in the facility was not considered.

Variations in lux intensity, noise and vibration measurements were not considered, as the application used for measuring these parameters is dependent on phone camera features and different iPhone models were used. Furthermore, proportions of tank size relative to animal length were not analyzed. Other factors that can substantially impact outcomes are the type of tracking software used. A recent study demonstrated discrepancies in tracking accuracy and behavioral quantification across different software, emphasizing the importance of selecting appropriate tools for data analysis^84^. Although we did ask whether the laboratories used automated tracking software, we did not ask for specific software. Likewise, we also did not account for the microbiome composition in zebrafish colonies.

Finally, we did not test the effect of validated drugs across laboratories as positive controls in this experiment. For example, many studies have consistently found differences in novel tank test performance after treatment with anxiolytic or anxiogenic compounds^16,18,26,28,29^. Future studies incorporating standardized drug treatments could further clarify how inter-laboratory variations influence behavioral and drug-induced effects in the novel tank test.

## Conclusions and practical implications

The present multi-laboratory study aimed to understand the variability in adult zebrafish behavior across different laboratories, focusing on the novel tank test, a common measure of anxiety-like behavior. Our findings indicate that, while laboratory-specific conditions and settings have a minor impact on the outcomes, the time spent in the test is critical across all laboratories. Key predictors of behavior included sex, feed type, facility size, noise and using a separate room to test the animals. Transporting fish to a different room increased anxiety-like behaviors, with the effect growing stronger over time. Noise was shown to increase anxiety-like behaviors; however, this was also time-dependent.

The present large-scale global collaborative study further supports the importance of considering housing and husbandry factors in behavioral research. While laboratory-specific conditions have a relatively minor impact, factors such as sex, stocking density, feed type and testing environment substantially contribute to anxiety-like behaviors in zebrafish. These findings highlight the need for standardized protocols to optimize zebrafish husbandry and ensure robust, reproducible experimental results. In practical terms, researchers should carefully control and report sex differences at the very least. However, it is likely that efforts to maintain optimal stocking densities around 5 fish/L, provide nutritionally rich and continuous feed sources, and minimize the stress associated with transporting fish to new environments would also be prudent. Furthermore, attention should be given to the personnel interactions within housing facilities. By addressing these factors, researchers may be able to improve the reliability and consistency of behavioral studies in zebrafish, ultimately enhancing the validity of this model for studying neuropsychiatric disorders and other conditions.

## Methods

### Animals, experimental design and ethics

All laboratories carried out the work following local and/or national ethical approvals as follows.

#### Parker laboratory (C.H., A.C. and M.P.)

All experiments were carried out following local ethical approval from the University of Portsmouth Animal Welfare Ethical Review Board (AWERB) and under UK Home Office Project Licence PP8708123.

#### Wong laboratory (R.Y.W. and J.C.)

All procedures and experiments were approved by the Institutional Animal Care and Use Committee of the University of Nebraska at Omaha (17-070-09-FC).

#### Medan laboratory (V.M. and L.M.M.)

All procedures and experiments were approved by the Institutional Animal Care and Use Committee of the University of Buenos Aires (P152).

#### Dynamical Systems laboratory (G. Lombardelli and M.P.)

All experiments and procedures were approved by the University Animal Welfare Committee of New York University (protocol 13-1424).

#### Piato laboratory (M.G.-L., L.M.B., S.M.P. and A.P.)

All procedures and experiments were approved by the Animal Ethics and Research Committee at the Universidade Federal do Rio Grande do Sul (UFRGS, protocol 42179).

#### Kenney laboratory (B.D.F. and J.W.K.)

All experiments were approved by the institutional animal care and use committee of Wayne State University, protocol 21-02-3238.

#### Lardelli laboratory (E.G., K.B. and M.L.)

All experiments were performed under the auspices of The University of Adelaide Animal Ethics Committee (permits S-2017–089 and S-2017–073)

#### Laboratory of Experimental Neuropsychobiology (J.V.B., C.M.R. and D.B.R.)

All procedures were approved by the Institutional Animal Care and Use Committee at the Federal University of Santa Maria (process 7412110722).

#### Luchiari laboratory (J.R.T. and A.C.L.)

All experiments were approved by the Ethics Committee on the Use of Animals of the Federal University of Rio Grande do Norte (permit CEUA 358.036-2023).

#### Brennan laboratory (C.H.B., A.L. and X.W.)

All procedures were carried out under licence in accordance with the Animals (Scientific Procedures) Act, 1986 (UK) and under guidance from the Local Animal Welfare and Ethical Review Board at Queen Mary University of London.

#### Laboratory Animal Science group (A.M.V.)

All procedures were approved by our institutional committee (Animal Welfare and Ethics Review Body of the i3S; 2021–24) and by the National authority Direção Geral de Alimentação e Veterinária (DGAV; permit 19606/24-S).

#### Bonan laboratory (C.D.B., S.A. and B.D.P.)

All experiments were approved by the Ethics Committee on the Use of Animals at Pontifícia Universidade Católica do Rio Grande do Sul (permit number CEUA 10617).

#### Rutherford laboratory (E.O.G., M.L.P.-R. and C.A.S.)

All experiments were approved by the Institutional Animal Care and Use Committee at Indiana University School of Medicine – Northwest (protocol NW-49).

#### Hindges laboratory (E.R. and R.H.)

All procedures were carried out in accordance with the Animals (Scientific Procedures) Act 1986 under license from the UK Home Office (PP7266180).

#### Kalueff laboratories

Novosibirsk laboratory (T.G.A., E.V.N. and M.A.K.): all experiments were approved by the local ethical committee at the Scientific Research Institute of Neurosciences and Medicine (Novosibirsk, October 2023). St. Petersburg laboratory (D.S.G. and A.V.K.): all experiments were approved by the local ethical committee at Almazov Medical Research Center (St. Petersburg, October 2023). Sochi laboratory (T.O.K. and A.V.K.): all experiments were approved by the local ethical committee at Sirius University of Science and Technology (Sochi, October 2023). Ural laboratory (S.L.K, M.A.G. and A.V.K.): all experiments were approved by the local ethical committee at Ural Federal University (URFU, October 2023).

#### Translational Psychiatry laboratory (E.P.R., G. Lodetti and E.R.D.)

All procedures followed the National Institute of Health Guide for Care and Use of Laboratory Animals. The Ethics Committee of the University of Southern Santa Catarina (UNESC) approved the protocol under the number 74/2023.

#### Molecular Pharmacology and Medicinal Chemistry laboratory (P.I.-V., A.F.-C. and L.M.M.-D.)

Animal study protocol was approved by the Institutional Review Board (Ethics Committee) of Universidad de La Frontera (protocol code 056-23 approved on 2 June 2023).

See full details of the questionnaires used in the present study in the Open Science Framework (OSF).

A total of *N* = 488 experimentally naive adult zebrafish (240 females and 248 males), ranging from 3 to 7 months post-fertilization, were tested in 20 different laboratories for 5 min in the novel tank test. A sample size of *n* = 12 per sex was used following initial effect size and power calculations using G*Power^85^ from three original novel tank test papers^26,28,39^ with a calculated power of 0.95 and required sample size of 4. However, we asked each laboratory to aim to test 12 male and 12 females to account for the potential (thus far unknown) variance between laboratories. A variety of zebrafish wild-type genotypes were included (including AB, TU, SF or commercially acquired wild types). The main behavioral parameters collected for data analysis, including an in-depth analysis of housing conditions and experimental setups, are available on the OSF linked to this Article. No animals were removed from the data analysis.

### Variables

We examined variables that may affect zebrafish novel tank performance, including the test apparatus and tested animals, such as tank size and animal age, because they both can affect zebrafish novel tank test^28,43^. Moreover, gentle handling and fast transfer to the novel tank was considered, to account for possible effects of human interaction and stress in fish, by asking laboratories to describe their fish handling processes in detail^86^. We also assessed whether behavioral testing was carried out in a separate room, as this can reduce environmental variability and external disturbances, providing more controlled conditions^39,51^. Automatization was also considered by asking researchers whether behavior was recorded automatically or manually. The videos were analyzed per minute for 5 min.

The physical structure of the tank environment was assessed by recording the number of racks in the holding facility, whether the rack was static or circulating, and the total number of fish in the facility. Static racks are likely to provide a different environmental experience compared with circulating racks, which can influence water flow, noise and/or vibration and oxygenation, potentially affecting fish movement and comfort^87^. Temperature of the holding room and temperature of the water in the housing room were included as both ambient and water temperatures directly affect fish physiology and behavior, potentially leading to changes in activity levels and stress responses^88–90^. The pH level of the housing tank was measured as a numerical predictor because the acidity or alkalinity of the water can substantially affect fish health and behavior^91,92^. The density of fish per liter of water in the housing tank was also considered, as population density can impact stress levels and social behaviors, thereby affecting diving performance^65–67,93,94^.

Environmental enrichment data were included because environmental enrichment can provide stimulation and reduce stress, potentially impacting zebrafish behavior and well-being^72,95–97^. Lighting conditions are likely to be substantially important for a range of behavioral differences, and thus light levels were measured from various angles, including lux levels at the top, base, front and rear of the housing tank (‘Light Meter’, https://apps.apple.com/us/app/lux-light-meter-pro/id1292598866). Light intensity and direction can influence fish visibility and orientation^98^ as well as circadian rhythms and sleep^99^. Vibration levels at the top and bottom of the tank were also recorded, as vibrations can impact fish behavior and stress levels, with different effects depending on where the vibrations occur (‘Vibrometer’, https://apps.apple.com/us/app/vibration-meter-seismograph/id1137580201)^100–102^. Noise levels in decibels (‘Decibel X’, https://apps.apple.com/us/app/decibel-x-db-sound-level-meter/id448155923) in the housing room were also included in our analyses because noise can be a significant stressor for fish, potentially impacting their behavior and physiology^95,101,102^. Finally, the color of the floor and walls in the room and the color of the housing tanks were measured, as these factors can influence the ambient light environment and the visual comfort of the fish^58^.

We included several dietary factors, including whether the fish were fed brine shrimp (artemia), flake food, bloodworm, rotifers or pellet food, as diet can impact fish health and behavior^73,103^. Finally, the frequency of feeding per day was recorded, as feeding schedules can influence activity levels, stress and routine behaviors^80,104^. Finally, we included data on some human factors, including the number of laboratory members working with the fish, the number of personnel daily entries to the fish holding room per day, the average age of the personnel and whether the personnel were male or female. These factors might correlate fish experience with humans and handling techniques, influencing fish stress and behavior differently^86,105^.

### Procedure

Through personal connected networks, we invited active laboratories around the world that perform adult zebrafish behavioral studies to take part in the study. An interactive global map highlighting each of the universities involved in this collaborative work was produced using a custom Python code using the Folium library for geographic visualization. The code as well as a link to the interactive version of the map is available (https://osf.io/4chwt/?view_only=024aa4208a83420c8fa38e2e0c64943al).

### Analysis and statistics

A one-way ANOVA was performed to evaluate the mean effects across laboratories on ‘distance traveled (cm)’, ‘time spent in the top of the tank (s)’ and ‘number of top entries’ followed by a Dunnett’s post-hoc test comparing each laboratory with the average, to evaluate inter-laboratory difference. Dunnett’s test was selected as it allows comparisons between each laboratory and a standardized reference rather than conducting all possible pairwise comparisons. Importantly, the average was calculated by generating 24 samples that fell into the values of the 20 laboratories and considered maximum data deviation for each parameter. Next, to evaluate potential sex effects on three parameters assessed in this work, we analyzed the effect size of female × male differences, and these effect sizes were represented through forest plots. Effect sizes were calculated using the standardized mean difference approach. Specifically, we used Cohen’s *d* to measure the effect size for each study included in the analysis. The weights were calculated as the inverse of the variances of the effect sizes and CIs were calculated using the effect size s.e.m. × 1.96 for a 95% confidence level. Following heterogeneity analysis, which resulted in moderate heterogeneity (*I*^2^), the overall average effect size in the forest plots was calculated using a random-effects model with the DerSimonian and Laird approach. This approach was chosen to account for the variability both within and between studies. Distance traveled and exploratory parameters were chosen for the analysis of sex effect size owing to previous studies showing that males travel more than females, and females can show higher anxiety-related responses^16,55^.

To evaluate variables that can influence novel tank behavior, mixed-effects models were fit to extract residuals for the two primary response variables, ‘time spent in the top of the tank (s)’ (time top) and the ‘number of top entries’ (top entries). The model structure included random effects for Lab ID nested within Fish ID. This allowed us to account for the hierarchical structure of the data, ensuring that variability at the Lab ID and Fish ID levels was appropriately modeled. The residuals from these models were then used as response variables in subsequent analyses.

In high-dimensional datasets such as this one (that is, datasets with a large number of variables relative to the number of observations), the extensive number of predictors can lead to challenges including overfitting, excessive complexity and, critically, difficulties in identifying the most important predictors. High-dimensional data therefore require specialized statistical methods, such as Lasso regression, that can perform variable selection and regularization to build more robust and interpretable models^106^. First, to explore the fixed effects and their interactions, we created several design matrices based on different fixed effects formulas, including main effects, interactions of main effects with sex and time, and finally a combined sex × time interaction model. Lasso regression models were then fit using the resulting design matrices^106^. Cross-validation was performed to determine optimal lambda values to control the regularization strength of the Lasso models, to prevent overfitting and to ensure that the models generalized well to new data^106^. We next performed bootstrapping with 1,000 iterations to estimate the variability of the coefficients generated from each Lasso model. Using the bootstrapped results, 95% CIs were calculated for the coefficients of the Lasso models. Significant predictors were identified on the basis of the coefficients and their CIs. Any predictors for which CIs did not include zero were considered significant. Finally, deviance explained by each model was calculated to assess model fit, and AIC values were computed for each Lasso model to evaluate model parsimony, thus balancing model fit and complexity^107^. All data analysis was carried out in RStudio version 4.4.1 (https://osf.io/4chwt/?view_only=024aa4208a83420c8fa38e2e0c64943a) using several R libraries^108^. Data manipulation and preprocessing were conducted with the dplyr and readxl packages, which facilitated the handling of categorical variables and missing data. To check the distribution and identify outliers in our continuous variables, we used the ggplot2 library for creating histograms and boxplots^109^. Mixed-effects models were fit using the lme4 package to account for random effects, extracting residuals for further analysis. Specifically, we fit mixed-effects models to the time in the top (s) and number of top entries variables, incorporating random effects for Fish ID nested in Lab ID. We then applied Lasso regression using the glmnet package. A design matrix was created for the predictors, and cross-validation was performed to determine the optimal lambda value for the Lasso regression models. Separate Lasso regressions were conducted for the residuals of time in the top (s) and number of top entries to identify significant predictors. To ensure the robustness of our findings, we performed bootstrapping using the boot package, which provided 95% CIs for our coefficient estimates. Functions were defined to fit Lasso models on bootstrap samples and extract coefficients, and 1,000 bootstrap iterations were performed for both response variables. We extracted significant predictors based on nonzero coefficients. Finally, to validate the models, we refit the original LMMs with the predictors that emerged from the Lasso regression models.

### Reporting summary

Further information on research design is available in the Nature Portfolio Reporting Summary linked to this article.

## Online content

Any methods, additional references, Nature Portfolio reporting summaries, source data, extended data, supplementary information, acknowledgements, peer review information; details of author contributions and competing interests; and statements of data and code availability are available at 10.1038/s41684-025-01548-x.

## Supplementary information

Supplementary Information Supplementary Figs. 1–6 and Table 1.

Reporting Summary

## Data Availability

All raw data and analysis files for this study are available via OSF at https://osf.io/4chwt/?view_only=024aa4208a83420c8fa38e2e0c64943a
