# Supplementary material for: Housing and husbandry factors affecting zebrafish novel tank test responses: a global multi-laboratory study
Source: Lab Anim (NY). 2025 May 26;54(6):156–64. doi: 10.1038/s41684-025-01548-x (PMC12129812; doi:10.1038/s41684-025-01548-x)
Supplement: Supplementary file 1 — Supplementary Figs. 1–6 and Table 1. [file 41684_2025_1548_MOESM1_ESM.pdf]

lab animal

https://doi.org/10.1038/s41684-025-01548-x

Housing and husbandry factors affecting zebrafish novel tank test responses: a global multi-laboratory study

In the format provided by the authors and unedited

Supplementary Materials

Supplemental Figures

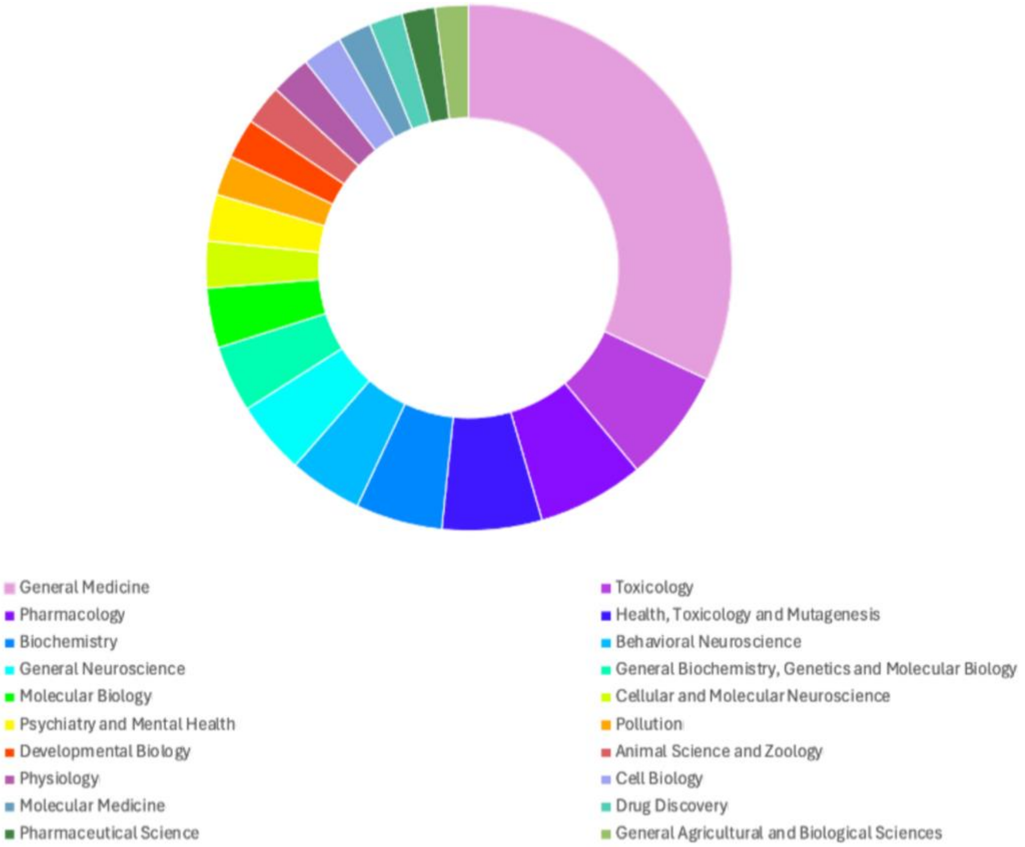

Fig. S1. Summary of the different fields of scientific research that have publications using the zebrafish novel tank behavioral assay. This demonstrates the wide and varied use of the assay regardless of the field of study. Data obtained from the PubMed search described previously.

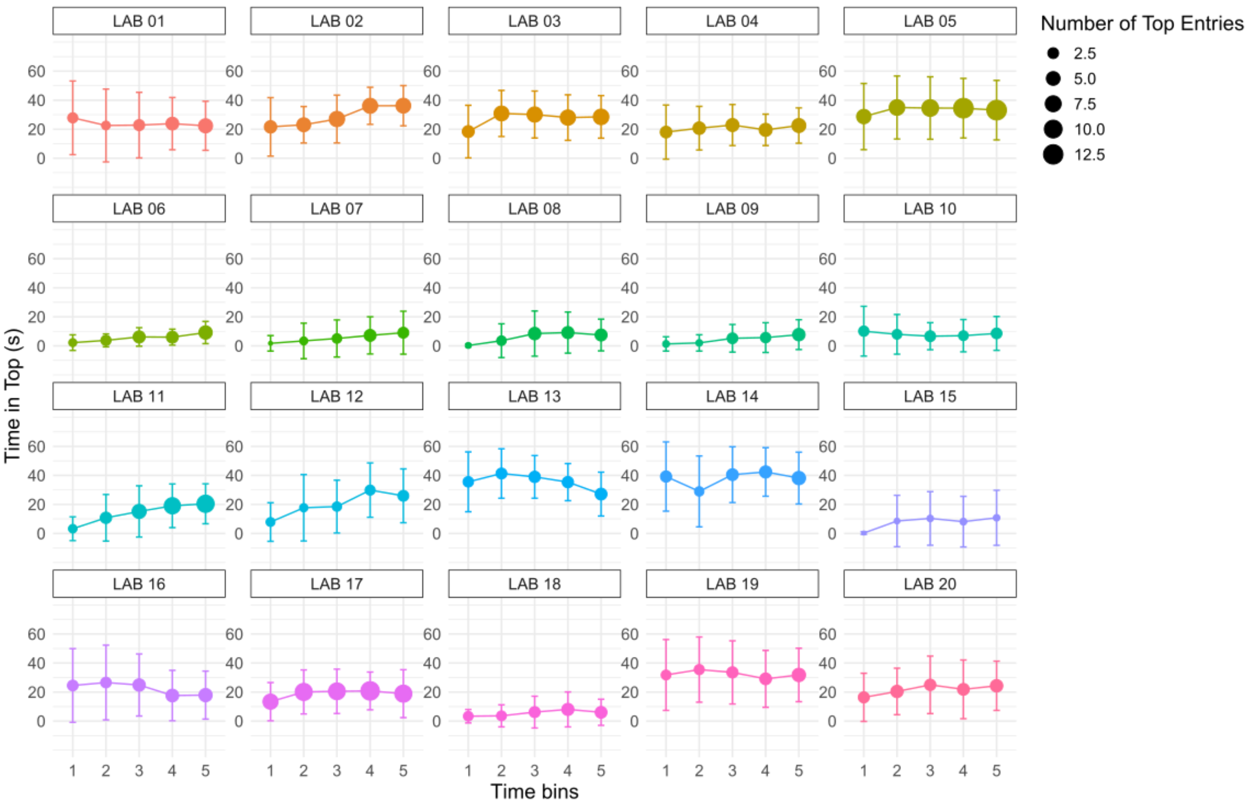

Fig. S2. Average time spent at the top of the tank across time for each laboratory. Circles represent the number of entries in the top zone and are proportional to the average number of entries per time bin (1 minute time bins). Data represented as mean ± SD.

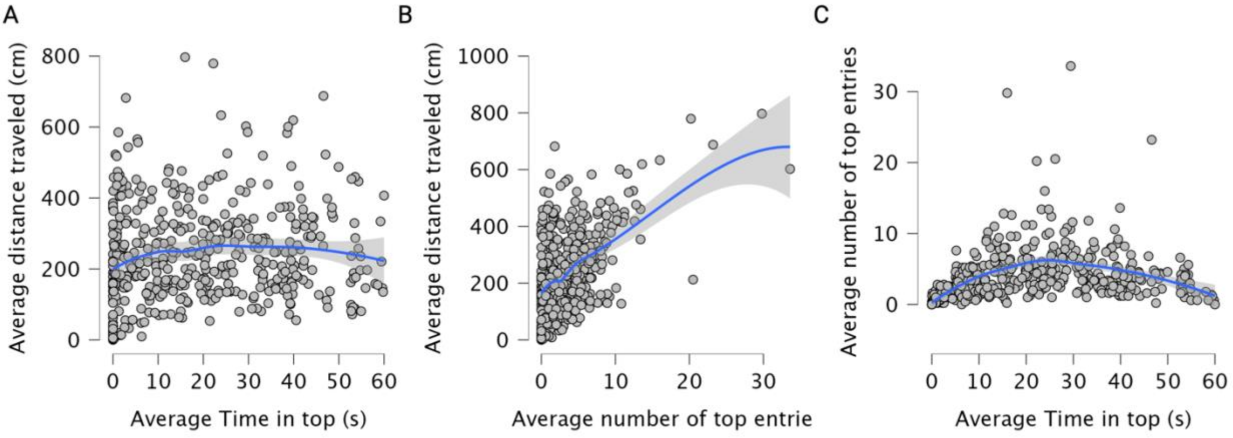

Fig. S3. Scatter plots representing correlations for the variables (A) number of top entries with average time in top (s), (B) distance travelled (cm) with number of top entries and (C) distance travelled with time in top (s).

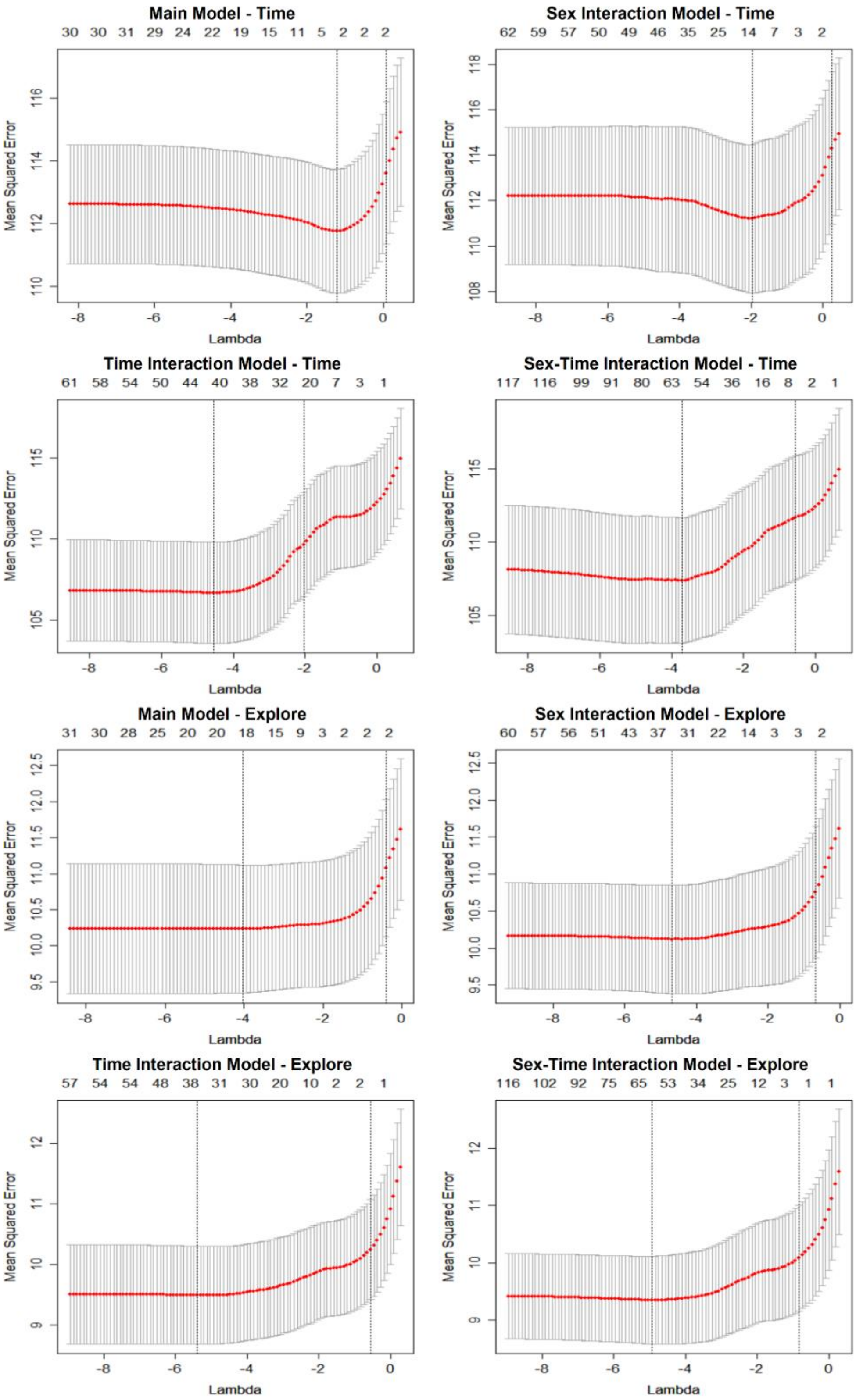

Fig. S4. Cross-Validation Results for Lasso Regression Models

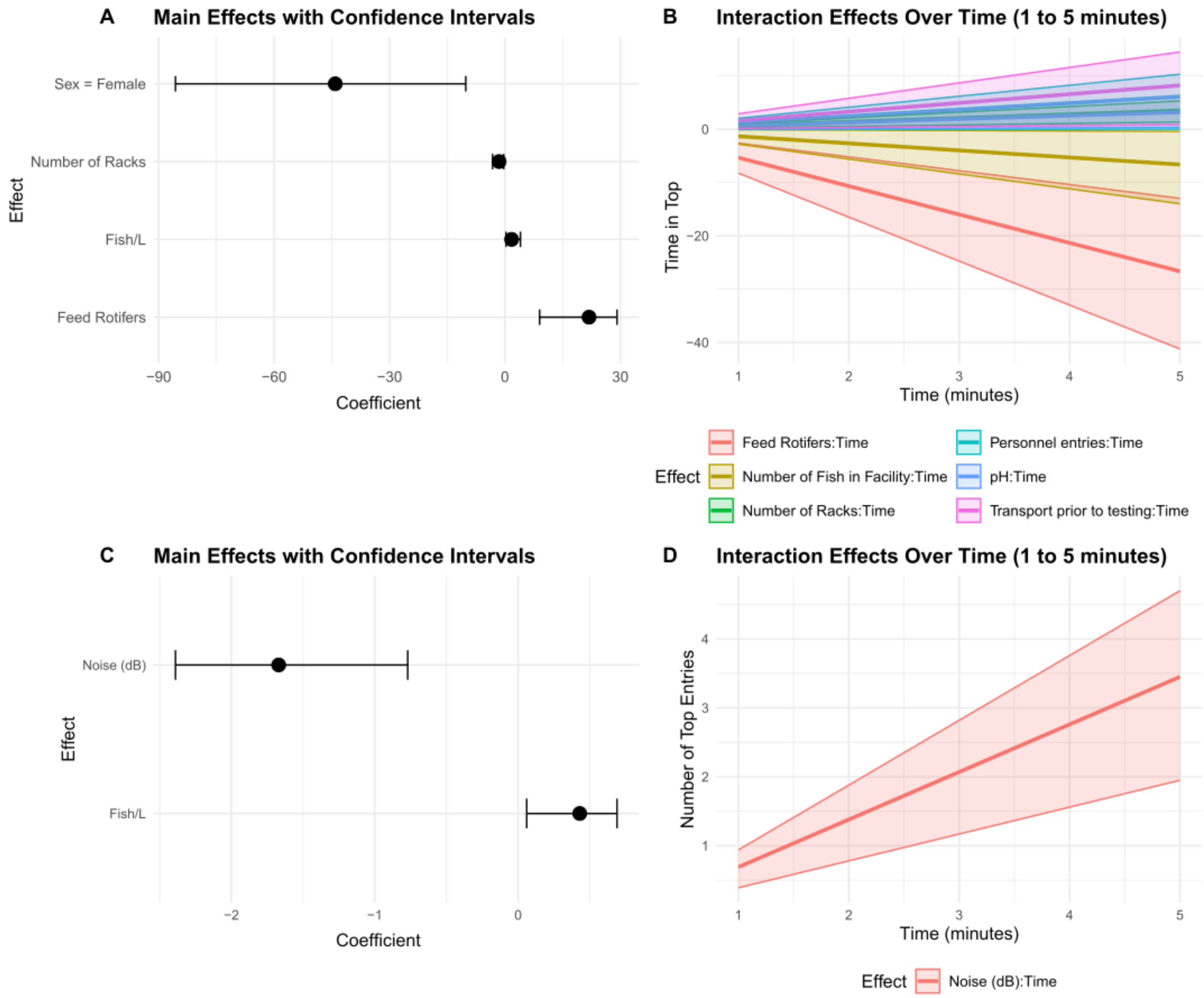

Fig. S5: Main Effects and Confidence Intervals for Significant Predictors (A – time in top; C – top entries). Interaction Effects Over Time for Significant Predictors (B – time in top; D – top entries)

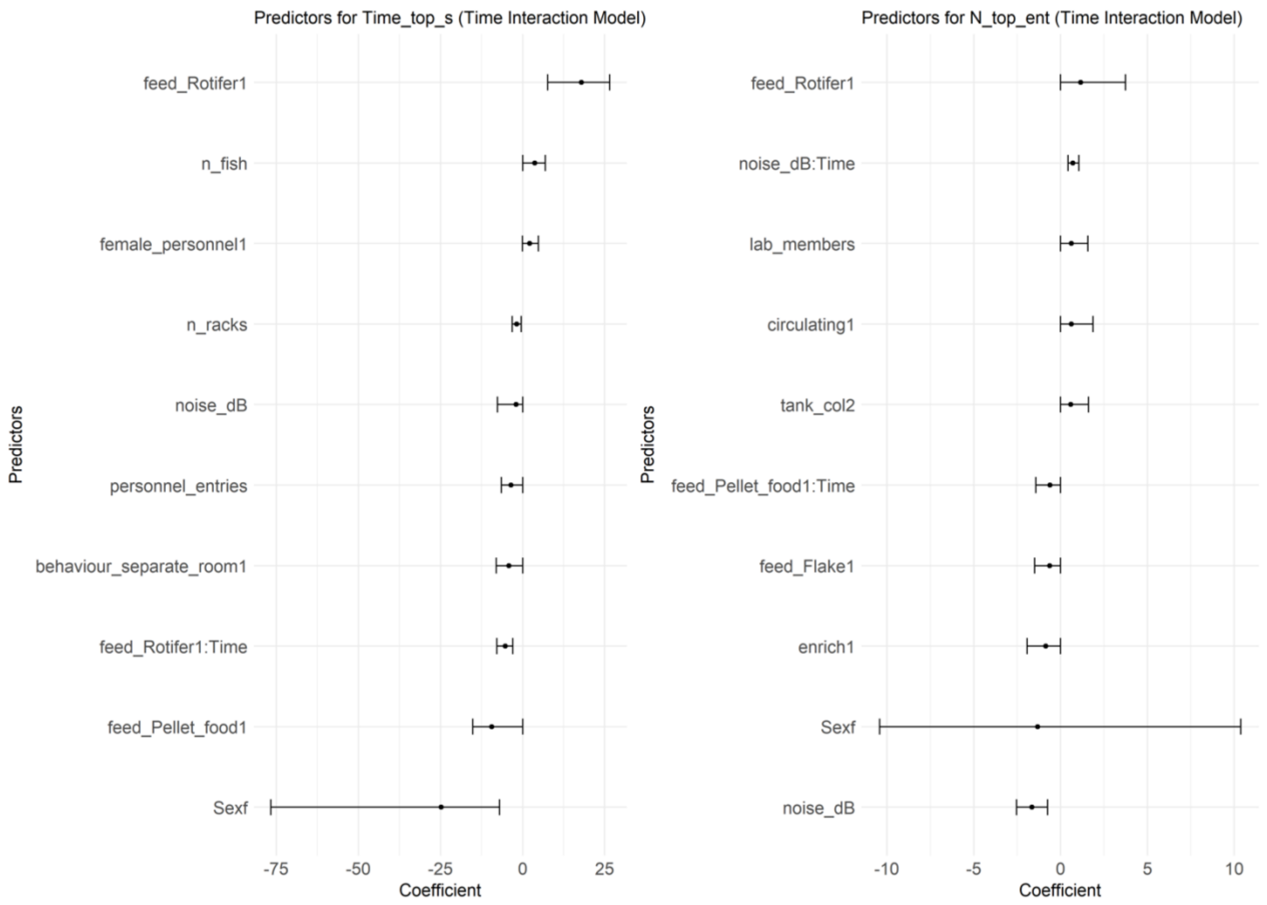

Fig. S6. Predictors for time in top and number of top entries using the Time interaction model represented as coefficient and confidence intervals.

Table S1. List of parameters which we recommend are reported on in all future novel tank test (NTT) experiments.

**Parameters to be reported on**
Acclimation period
Breeding status
Colors in NTT
Dimensions of NTT
Experimenter’s age, sex, seniority and how often they work with the fish
Feeding parameters
Habituation period
Housing colors
Lighting (Lux) – housing and for NTT

Noise levels (dB) – housing and for NTT
Sex split
Stocking density
Strain
Tank enrichment
Time of day of NTT
Tracking software (automated vs manual)
Water changes in between NTT
Water chemistry
Where NTT is performed

27
